# An Unusual Encounter: Microfilaria Incidentally Detected in the Bone Marrow Aspirate of a Chronic Kidney Disease Patient

**DOI:** 10.7759/cureus.59808

**Published:** 2024-05-07

**Authors:** Prima S Lakra, Jitendra K Sinha, Ujjawal Khurana, Ashwani Tandon, Rajnish Joshi

**Affiliations:** 1 Pathology and Laboratory Medicine, All India Institute of Medical Sciences, Deoghar, IND; 2 Pathology and Laboratory Medicine, All India Institute of Medical Sciences, Bhopal, IND; 3 Internal Medicine, All India Institute of Medical Sciences, Bhopal, IND

**Keywords:** chronic kidney disease (ckd), wuchereria bancrofti, w. bancrofti, microfilaria, kidney disease, filariasis, bone marrow

## Abstract

Lymphatic filariasis is endemic in a few states of India and is one of the most common public health concerns. *Wuchereria bancrofti* (*W. bancrofti*) is the most common parasite that causes lymphatic filariasis in India. Microfilariae have been commonly found in the peripheral blood and body fluid, as well as demonstrated in fine needle aspirates (FNA) and bronchial cytology. They have been rarely reported in bone marrow aspirates. Due to the nocturnal periodicity of *W. bancrofti*, it may be missed in peripheral blood during the day. Though peripheral eosinophilia is a presenting feature of filariasis, it may be absent in the majority of cases, as in this case. We report an incidental finding of *W. bancrofti* in the bone marrow aspirate of a 72-year-old male who had chronic kidney disease.

## Introduction

Lymphatic filariasis is endemic in a few areas of India and is one of the problems related to public health. In India, two species of *Wuchereria bancrofti *(*W. bancrofti*)* *and *Brugia malayi* are more commonly present [1]. It is a roundworm nematode under the Filarioidea type of infection. Lymphatic filariasis causes disability and is one of the causes of social stigma commonly affecting the legs and genitals in males [2]. Microfilariae are evident in fine needle aspirate (FNA) smears from diverse locations such as the thyroid, breast, and subcutaneous nodules, alongside cervical scrapes, bronchial washings, and various body fluids, in addition to blood and lymph node aspirates [3].

It is very rare to find microfilaria in bone marrow aspirates, with few cases reported of microfilaria being associated with hematological conditions like cytopenia, hypoplasia of the bone marrow, and myelofibrosis [4-5]. We report an incidental finding of microfilaria in a bone marrow aspiration sample of a 72-year-old male who was being evaluated for plasma cell dyscrasia.

## Case presentation

A 72-year-old male reported to a medicine clinic, with the chief complaint of low back pain for three months. The patient had a medical history of diabetes mellitus, chronic kidney disease, and chronic cystitis. The patient also had a dorsolumbar collapse. On examination, the patient had pallor, pedal edema, and no organomegaly. His hematological parameters showed a hemoglobin of 8.8 g/dl with a mild increase in leucocyte count. His other lab values were as follows: total leucocyte count: 13,360/µl, neutrophils: 74%, lymphocytes: 18%, monocytes: 05%, eosinophils: 33%, and basophils: 0%.

On the peripheral smear, the red cells were normocytic and normochromic. Platelets were adequate (2.6 lakh/cumm). The initial peripheral blood smear collected in the morning showed no hemoparasite. A bone marrow examination was performed to rule out plasma cell dyscrasia. Renal function tests revealed increased serum creatinine and urea levels (1.66 mg/dl and 59.62 mg/dl, respectively). Urine examination showed the presence of blood (1+), leucocytes (2+), and protein (2+). Urine microscopy showed the presence of granular casts and bacteria. Liver function was normal.

**Figure 1:**
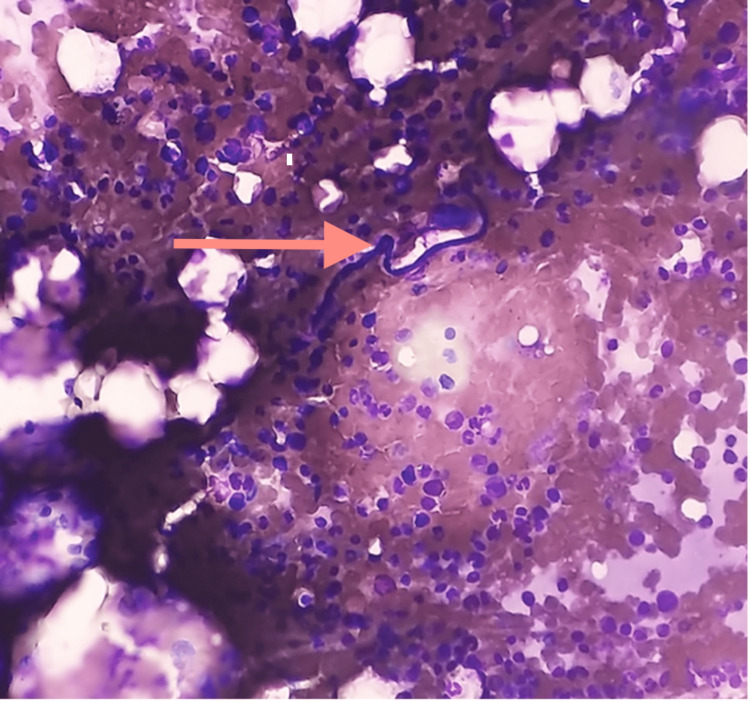
Bone marrow aspirate showing microfilaria (Giemsa stain, 10x)

**Figure 2:**
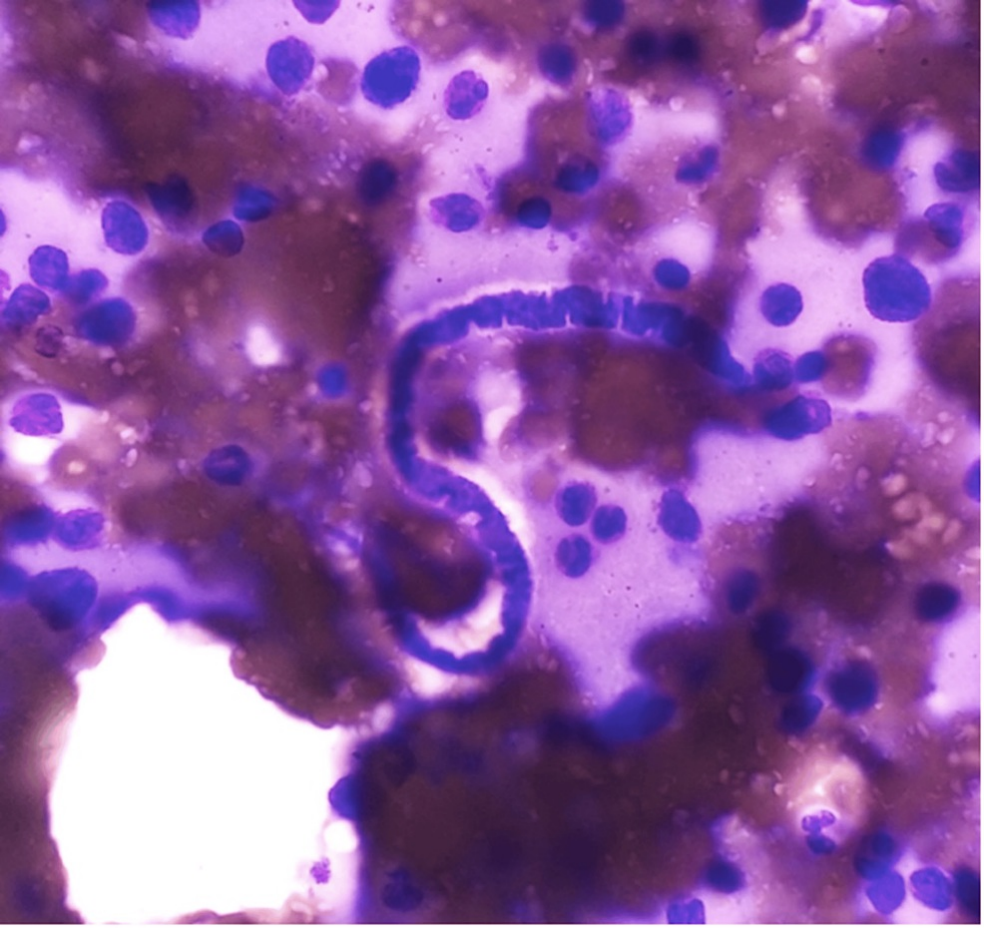
Bone marrow aspirate showing curved, pointed-tail microfilaria (Giemsa stain, 100x)

Since the patient was initially being evaluated for plasma cell dyscrasia, bone marrow studies were recommended by the treating physician. Bone marrow aspiration was reported as normocellular marrow with a myeloid-erythroid ratio of 3.5:1. Erythropoiesis was normoblastic, with myelopoiesis being adequate. There was a mild increase in megakaryopoiesis. Marrow smears also showed microfilaria, which were curved, cephalic spaces sheathed with a pointed tail, with nuclei not reaching up to the tip (Figures 1, 2). The morphology was that of *W. bancrofti*. 

**Figure 3:**
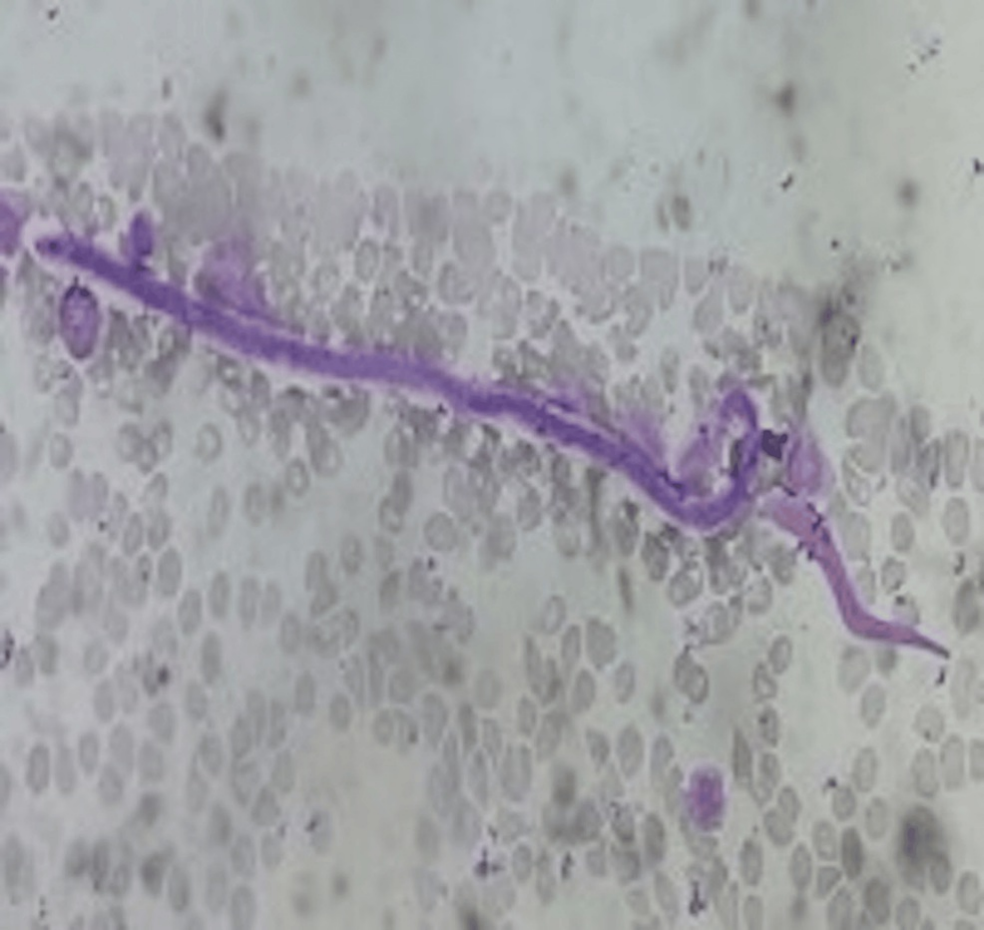
Peripheral smear showing microfilaria with a pointed tail (Leishman stain, 40x)

There was no increase in plasma cells (2%). A diagnosis of normocellular marrow with the presence of *W. bancrofti* was made on aspirate slides. A bone marrow biopsy showed no presence of microfilaria. Following the marrow finding, the clinician was advised to send a blood sample at midnight, which showed *W. bancrofti* in the peripheral smear (Figure 3). 

## Discussion

Filariasis is one of India’s health concerns; it is endemic. In India, the highest endemicity is in Bihar (over 17%), followed by Kerala and Uttar Pradesh. Madhya Pradesh has a low endemicity of 3% [6]. In our case, the patient was a native of Madhya Pradesh, which has an endemicity of 3%.

*Wuchereria bancrofti* is one of the important causes of lymphatic filariasis. The most common presentation of lymphatic filariasis is lymphangitis, elephantiasis, and peripheral blood eosinophilia. Absence of peripheral eosinophilia is not uncommon, as observed in prior case reports [4, 5, 7 ]. The absence of peripheral blood eosinophilia in these cases may be due to oxidative stress associated with filariasis, which can cause altered or changed immune responses [8].

*Wuchereria bancrofti *is mostly found in peripheral blood smears at night. In our case, it was not detected in the earlier samples but was seen in the midnight sample. Anaemia and pancytopenia were the other frequent peripheral blood findings [5, 7]. In the indexed case, the patient did not have pancytopenia but had anemia. The patient had a slightly high total leucocyte count, which can be attributed to chronic cystitis. The first documented case of microfilariae in bone marrow aspirates was by Pradhan et al. in 1976 [7]. 

Microfilariae may enter various organs and body fluids via peripheral blood. *Wuchereria bancrofti* has been demonstrated in aspirates with breast, thyroid, and forearm swelling [3]. There are cases in the literature where microfilariae have been associated with bone marrow hypoplasia [5]. *Wuchereria bancrofti *has been reported in cases with bone marrow metastasis as an incidental finding [4].

However, it is not yet clear how these microfilariae reach the bone marrow. As suggested by Pradhan et al., the microfilariae may migrate and enter the bone marrow by crossing the vessel wall, which can be due to their borrowing ability [7]. Sinha et al. reported microfilariae in bone marrow aspirates, presenting as pancytopenia [1].

The presence of microfilaria was an incidental finding in this case, as the patient was being evaluated for chronic kidney disease and plasma cell dyscrasia. The involvement of the kidney, manifesting as glomerulonephritis, hematuria, and proteinuria, is seen as associated mainly with microfilaremia [9, 10]. The indexed case had hematuria with proteinuria. Granular casts were also noted in urine microscopy. 

Damage of glomeruli by microfilariae may account for hematuria in some cases, but it is seen that deposition of immune complexes in the glomerular basement membrane is a more common cause of renal pathological changes in bancroftian filariasis [11].

Being from a low-endemicity region, the patient was not evaluated for filariasis, which may have led to chronic kidney disease and cystitis. The presence of eosinophilia in symptomatic and asymptomatic patients prompts the search for parasitic infection. But in this case, peripheral eosinophilia was absent, which led to undiagnosed filaria, which might have led to chronic conditions like kidney disease. Another reason for undiagnosed filariasis is the nocturnal periodicity of *W. bancrofti*. 

## Conclusions

Filariasis may not be suspected clinically in areas of low endemicity and, therefore, go undiagnosed. Peripheral eosinophilia was absent, which led to undiagnosed filaria. In the endemic areas, all the bone marrow aspirates should be screened for microfilariae to detect any asymptomatic carriers.
